# Tenuigenin promotes the osteogenic differentiation of bone mesenchymal stem cells in vitro and in vivo

**DOI:** 10.1007/s00441-016-2528-1

**Published:** 2016-11-14

**Authors:** Hua-ji Jiang, Xing-gui Tian, Shou-bin Huang, Guo-rong Chen, Min-jun Huang, Yu-hui Chen, Bin Yan, Sheng-fa Li, Jia-jun Tang, Hui-yu Zhao, Liang Wang, Zhong-min Zhang

**Affiliations:** 1 Department of Orthopedics, The Third Affiliated Hospital, Southern Medical University, 183 West Zhongshan Avenue, Guangzhou, 510282 Guangdong People’s Republic of China; 2 Department of Orthopedics, Huizhou First Hospital, Huizhou, Guangdong People’s Republic of China

**Keywords:** Osteoporosis, Tenuigenin, BMSCs, Bone parameters, WNT/β-catenin signaling

## Abstract

Osteoporosis, which is a systemic skeletal disease characterized by low bone mineral density and microarchitectural deterioration of bone quality, is a global and increasing public health problem. Recent studies have suggested that Tenuigenin (TEN), a class of native compounds with numerous biological activities such as anti-resorptive properties, exerts protective effects against postmenopausal bone loss. The present study aims to investigate the osteogenic effects of TEN on bone mesenchymal stem cells (BMSCs) in vitro and in vivo. Alkaline phosphatase (ALP) activity/staining, Alizarin red staining and the expression of osteogenic markers, including runt-related transcription factor 2, osterix, osteocalcin, collagen Iα1, β-catenin and glycogen synthase kinase-3β were investigated in primary femoral BMSCs from C57/BL6 mice cultured under osteogenic conditions for 2 weeks to examine the effects of TEN. An ovariectomized (OVX) mouse model was used to investigate the effect of TEN treatment for 3 months in vivo. We found that ALP activity, mineralized nodules and the expression of osteogenic markers were increased and WNT/β-catenin signaling was enhanced in vitro and in vivo. Bone parameters, including trabecular thickness, trabecular number and bone mineral density were higher in the OVX+TEN group than in control OVX mice. Our results suggest the therapeutic potential of TEN for the treatment of patients with postmenopausal osteoporosis.

## Introduction

Osteoporosis (OP) is a common and systemic skeletal disease characterized by low bone mineral density (BMD) and microarchitectural deterioration of bone quality that reduces bone strength, with a consequent increased risk of fragile fractures (van den Bergh et al. 2012). More than 200 million people worldwide are affected by osteoporosis, especially postmenopausal women and older men (Liu et al. 2015; Tao et al. 2016). Osteoporosis increases the frequency of fractures of the hip, spine and wrist in association with substantial morbidity and mortality and the direct costs of osteoporotic treatments appear to be rising dramatically concomitant with the increase in life expectancy (Li et al. 2016). Although hormone replacement therapy is the most common therapeutic approach for the prevention and treatment of postmenopausal osteoporosis, the Women’s Health Initiative reported that the health risks of hormone replacement therapy exceed its benefits (Yu et al. 2016). The use of bisphosphonates for the treatment of osteoporosis has also been reported (Bagan et al. 2016; Eriksen et al. 2014; Safer et al. 2016). However, the potential bone-forming agents in bisphosphonates are associated with serious side effects and may not yield the expected improvements in bone quality and bone union ratio (Luhe et al. 2008). Furthermore, the cost-effectiveness of their widespread or long-term use has been questioned. Despite the availability of an armamentarium of agents, finding the optimal agent remains a challenge (Demontiero et al. 2012). Therefore, it is desirable to identify better and safe anabolic agents for the treatment of osteoporosis.

Bone mass is controlled by continuous bone remodeling through osteoblastic bone formation and osteoclastic bone resorption. Abnormalities in bone remodeling can produce a variety of bone-decreasing disorders such as osteoporosis (Rodan and Martin 2000). During bone remodeling, the removal of old bone from the skeleton is performed by osteoclasts, which are derived from hematopoietic stem cells and the addition of new bone occurs through differentiation/mineralization by osteoblasts, which are derived from mesenchymal stem cells (Boyle et al. 2003; Harada and Rodan 2003). If the balance between bone formation and bone resorption is disturbed and the rate of bone formation is lower than that of bone resorption, adult skeletal diseases can result in osteoporosis.

Bone mesenchymal stem cells (BMSCs) are composed of progenitor and multipotent skeletal stem cells and can differentiate into osteoblasts, osteocytes, adipocytes and chondrocytes in vitro (Ma et al. 2015). At present, it is understood that all osteoblasts are derived from BMSCs (Zhang et al. 2016). As BMSCs can differentiate into skeletal cell phenotypes (Bianco et al. 2006), they are the most promising and thoroughly investigated population of stem cells in osteoporosis research.

Tenuigenin (TEN), the chemical structure of which has been elucidated, is the active component of the root of the Chinese herb *Polygala tenuifolia*. It has a variety of biological activities, including anti-apoptotic, anti-oxidative and anti-inflammatory effects (Liang et al. 2011; Sun et al. 2007). A recent report suggested that TEN is a potential drug for the treatment of osteoporosis by suppressing osteoclastogenesis (Yang et al. 2015). Osteoblast differentiation and osteoclastogenesis are two important phases of bone remodeling; however, whether TEN affects the osteogenic differentiation of BMSCs in vitro and bone formation in vivo remains unknown.

In the present study, we investigate the osteogenic activity of TEN and its underlying mechanism in vitro and in vivo. Our results indicate that TEN induces the differentiation of BMSCs into osteoblasts by activating WNT/β-catenin signaling.

## Materials and methods

### TEN preparation

**Fig. 1:**
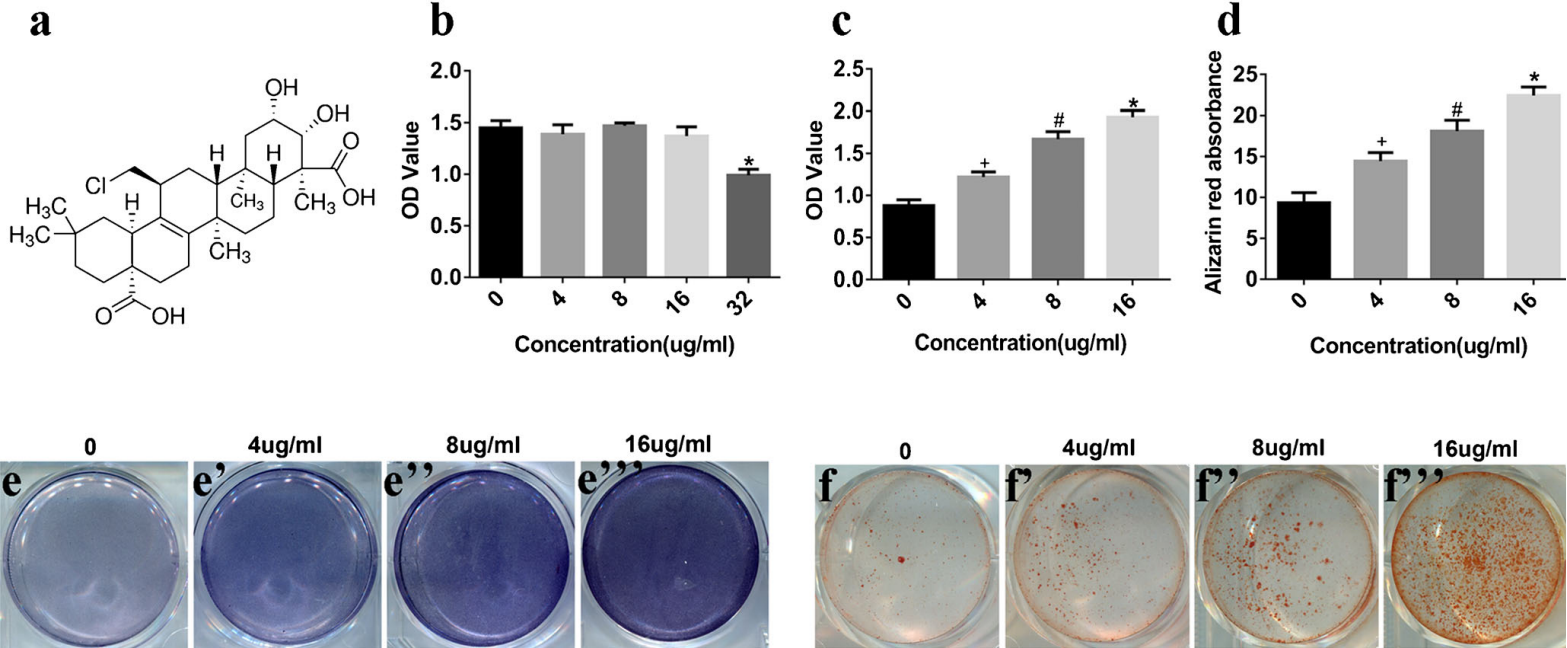
TEN increased osteogenesis in BMSCs. **a** Structure of TEN. **b** BMSCs was treated with various concentrations of TEN (4, 8, 16 μg/ml) for 14 days and cell viability was measured by using the CCK-8 assay. BMSCs were incubated with TEN (4, 8, 16 μg/ml) in osteogenic differentiation medium for 14 days, then ALP activity (**c**), mineralized nodules (**d**), ALP staining (**e**–**e**”’), and Alizarin red staining (**f**-–**f”’**) were tested. ^+^ *P* < 0.05 versus group without TEN, ^#^ *P* < 0.05 compared with 4 μg/ml TEN, *P < 0.05 compared with 8 μg/ml TEN. *Column*s represent means ± SD from 3 independent experiments, each performed in triplicate

TEN (Fig. 1a) was obtained from Sigma–Aldrich (St Louis, MO, USA). A stock solution of TEN (10 mg/ml) was prepared in dimethyl sulfoxide (DMSO; Sigma–Aldrich) and stored at 2–8 °C. TEN stock was diluted in sterile phosphate-buffered saline (PBS) and PBS-DMSO (0.16 %) served as the control in cell culture experiments.

### Animals and drug treatment

A total of 30 female C57BL/6 mice aged 8 weeks and weighing 22 ± 3 g were provided by the Southern Medical University (Guangzhou, Guangdong, People’s Republic of China). The mice were assigned equally to each of three groups: sham, ovariectomized (OVX) and OVX+TEN. OVX+TEN mice received TEN (6 mg/kg/day) intragastrically every day for 3 months after ovariectomy. The sham and OVX groups received the same amount of normal saline. Ethical approval for this study was obtained from the Medical Ethics Committee of the Southern Medical University (2015-095). The mice were kept following the animal care guidelines of the Southern Medical University laboratory animal welfare and ethics committee charter.

### Cell culture and osteogenic differentiation

BMSCs were isolated from C57BL/6 mice (aged 4 weeks). Briefly, mice femurs were dissected free of surrounding soft tissue. The bone marrow was flushed with α-MEM (Gibco, Grand Island, NY, USA). The marrow content from four to six bones was plated in culture flasks containing BMSC growth media (α-MEM containing 10 % fetal bovine serum, 100 U/ml penicillin,100 mg/ml streptomycin sulfate; Sigma-Aldrich). Non-adherent cells were removed and adherent BMSCs were cultured and expanded for further experiments. Primary cells were used in the experiments prior to the third passage. Cell culture media were replaced every 3 days.

### Cell proliferation assay

Primary BMSCs were seeded in 96-well plates at a density of 3.13 × 10^4^ cells/cm^2^. After 2 days in culture, cells were treated with TEN at concentrations of 4, 8, 16 and 32 μg/ml for 14 days. Cell proliferation was assessed using the Cell Counting Kit-8 (CCK-8; Keygen Biotech, Nanjing, People’s Republic of China) colorimetric assay according to the manufacturer’s instructions. Absorbance was measured at 450 nm.

### ALP activity/staining assay

Cells were plated into six-well plates at a density of 2.08 × 10^4^ cells/cm^2^. Following treatment with TEN (4–16 μg/ml) and osteoblast differentiation for 14 days, the cells were washed twice with PBS, scraped into 500 μL of 10 mM Tris-HCl buffer (pH 7.6) containing 0.1 % Triton X-100, placed on ice and sonicated to lyse the cells. Protein concentrations in the lysates were determined using the Bradford protein assay. Alkaline phosphatase (ALP) activity in the cellular fraction was measured using a fluorometric detection kit (Nanjing Jiancheng Biotechnology, Nanjing, People’s Republic of China). A standard curve was created using *p*-nitrophenol as the standard and the ALP activity of each sample was calculated from optical density (450 nm) values. Parallel wells were stained for ALP. Cells were fixed in 4 % paraformaldehyde (Sigma-Aldrich) for 20 min at room temperature, washed, incubated with ALP staining buffer (NBT-BCIP; Sigma-Aldrich) at 37 °C for 30 min and washed with PBS to remove excess dye.

### Alizarin red assay

BMSCs (2.08 × 10^4^ cells/cm^2^) were plated and cultured in six-well plates with various concentrations of TEN (4–16 μg/ml) in osteogenic medium for 14 days. On day 14, the Alizarin red assay (Sigma-Aldrich) was performed to determine mineralization. Briefly, cells were washed with PBS and fixed with 4 % paraformaldehyde for 30 min at room temperature. Fixed cultures were incubated with 1 % Alizarin red for 30 min and washed with PBS to remove excess dye. Extracellular matrix mineral-bound stain was photographed under a microscope. For quantification, the bound staining was eluted with 10 % (wt/vol) cetylpyridinium chloride (Sigma-Aldrich) and the absorbance of the supernatants was measured using an automated microplate reader (Bio-Rad, Hercules, CA, USA) at 570 nm.

### Quantitative reverse transcription PCR (qRT-PCR) assay

Total RNA was isolated from BMSCs with the TRIzol reagent (Life Technologies, Grand Island, NY, USA) according to the manufacturer’s instructions after 2 weeks of osteogenic induction. Total RNA products were immediately converted to cDNA by reverse transcription (RT) using a PrimeScript RT reagent Kit with gDNA Eraser (TaKaRa, Dalian, China). Polymerase chain reaction (PCR) amplification was performed in a Chromo4 Four-Color Real-Time PCR Detection System (Bio-Rad) using a SYBRR Premix Ex Taq II (Tli RNaseH Plus) kit (TaKaRa). Primer sequences (Life Technologies) for each gene used in this study are shown in Table 1.

**Table 1:** Primer sequences used in RT-PCR and qPCR

Target gene	GenBank accession no.	Sequences (5’–3’)
GAPDH	NM_017008.4	Forward, CAGGGCTGCCTTCTCTTGTG Reverse, GATGGTGATGGGTTTCCCGT
RUNX2	NM_001145920.2	Forward, AATTAACGCCAGTCGGAGCA Reverse, CACTTCTCGGTCTGACGACG
OSX	NM_001037632.1	Forward, GCCTACTTACCCGTCTGACTTT Reverse, GCCCACTATTGCCAACTGC
OCN	NM_001037939.2	Forward, TCTATGACCTGCAGAGGGCT reverse,ATAGCTCGTCACAAGCAGGG;
Col1α1	NM_000088.3	Forward, AGTGGTTTGGATGGTGCCAA Reverse, GCACCATCATTTCCACGAGC
β-catenin	XM_006511927.1	Forward, CTGCAACGACCTGACTGGTA Reverse, GGCCATGTCCAACTCCATCA
GSK-3β	XM_006522425.1	Forward, AGAAGAGCCATCATGTCGGG Reverse, CCAAAAGCTGAAGGCTGCTG

### Western blot (WB) assay

Proteins isolated from six-well plates were subjected to SDS-polyacrylamide gel electrophoresis and transferred to polyvinylidene (PVDF) membranes (Sigma-Aldrich). Membranes were probed with rabbit polyclonal antibodies to runt-related transcription factor 2 (Runx2) (Cell Signaling Technology, Danvers, MA, USA), osterix (OSX) (Cell Signaling Technology), osteocalcin (OCN) (Santa Cruz Biotechnology, Santa Cruz, CA, USA), β-actin (Santa Cruz Biotechnology), β-catenin (Santa Cruz Biotechnology), glycogen synthase kinase-3β (GSK-3β) (Santa Cruz Biotechnology) and goat anti-rabbit secondary antibody (Santa Cruz Biotechnology). PVDF membranes were incubated with primary antibodies for 8 h at 4 °C and washed three times with TRIS-buffered saline with Triton X-100 (TBST; 5 min per wash). The secondary antibody was incubated with the membranes for 1 h at room temperature, followed by three washes with TBST (5 min per wash). Signals were revealed using an enhanced chemiluminescence kit (Cell Signaling Technology).

### Immunofluorescence staining

Cells were seeded into six-well plates containing glass cover slides at a density of 2.08 × 10^4^ cells/cm^2^ and grown to 95 % confluence. The cells received osteoblast differentiation medium containing TEN for 2 weeks. Following fixation in 4 % paraformaldehyde at room temperature for 30 min, the cells were washed three times with PBST (0.1 % Triton X-100 in PBS) and blocked in PBS-bovine serum albumin (1 % bovine serum albumin in PBS) for 1 h at room temperature. Cells were incubated with antibodies against collagen Iα1 (Col Iα1) (Abcam, Cambridge, MA, USA) for 1 h at room temperature. The samples were then washed three times in PBS for 10 min each and incubated with anti-rabbit immunoglobulin G (H+L), F(ab’) 2 Fragment Alexa Fluor 594 conjugated secondary antibodies (Cell Signaling Technology) for 1 h at room temperature. For nuclear staining, cells were counterstained with 4’,6-diamidino-2 phenylindole (DAPI, Cell Signaling Technology) for 5 min. Cells were examined using a laser confocal microscope (FV1000; Olympus Optical, Tokyo, Japan). Positive cells were evaluated using Image-Pro Plus software (Media Cybernetics, Rockville, MD, USA) to measure cellular fluorescence intensity. Cells with a fluorescence intensity ≥150 % of background were considered positive.

### Histological and immunohistochemical assay

Femur tissues dissected from the mice were fixed using 4 % paraformaldehyde in PBS at 4 °C for 24 h and then decalcified in 15 % ethylenediaminetetra-acetic acid (pH 7.2) at 4 °C for 14 days. The tissues were embedded in paraffin or optimal cutting temperature compound (Sakura Finetek) and sectioned at 2–5 μm. For histological analysis, the samples were stained with hematoxylin and eosin (HE) (Sigma-Aldrich). For immunohistochemistry, the sections were deparaffinized and briefly washed with PBS. This step was followed by incubation for 30 min in 3 % H_2_O_2_ to quench endogenous peroxidase activity. Appropriate primary antibodies anti-Runx2 (Cell Signaling Technology), anti-OSX (Cell Signaling Technology) and anti-OCN (Santa Cruz Biotechnology) were then applied overnight at 4 °C. After incubation with the primary antibody, sections were washed three times with PBS and incubated with goat-anti-mouse horseradish peroxidase-conjugated secondary antibodies (Cell Signaling Technology) for 1 h at room temperature. Immunostained sections were then incubated with DAB. Finally, the sections were dehydrated, mounted with coverslips and examined using an Olympus light microscope.

### X-ray micro CT assay

Long bones were collected from the mice, dissected free of soft tissue, fixed overnight in 4 % paraformaldehyde and analyzed by high-resolution micro-computed tomography (μCT 80; Scanco Medical, Bruttisellen, Zurich, Switzerland). We set the scanner at a voltage of 89 kV, a current of 112 μA and a scan thickness of 20 μm. Cross-sectional images of the proximal tibiae and femora were acquired to perform three-dimensional histomorphometric analyses of the trabecular bone. The analyses included various bone parameters, including trabecular thickness, trabecular number and trabecular BMD.

### Statistical analysis

Data are representative of at least three independent experiments. Each experiment was performed in triplicate. Data were graphed using GraphPad Prism software v.3.0. One-way analysis of variance followed by the Student’s *t* test was used to determine statistical differences. Error bars represent the standard error of the mean in the cell experiments and the standard deviation in the animal experiments.

## Results

### TEN has no toxic effects on the proliferation of BMSCs

Firstly, the toxicity of TEN was measured by the CCK-8 assay. BMSCs were treated with different concentrations of TEN (4-, 8-, 16- and 32 μg/ml) for 14 days. No alteration of cell viability was observed in BMSCs cultured in the presence of TEN at concentrations of 4, 8 and 16 μg/ml (Fig. 1b). The test suggested that TEN is not harmful to the proliferation of BMSCs at concentrations of 4, 8 and 16 μg/ml.

### TEN stimulates osteogenic differentiation of BMSCs in vitro

**Fig. 2:**
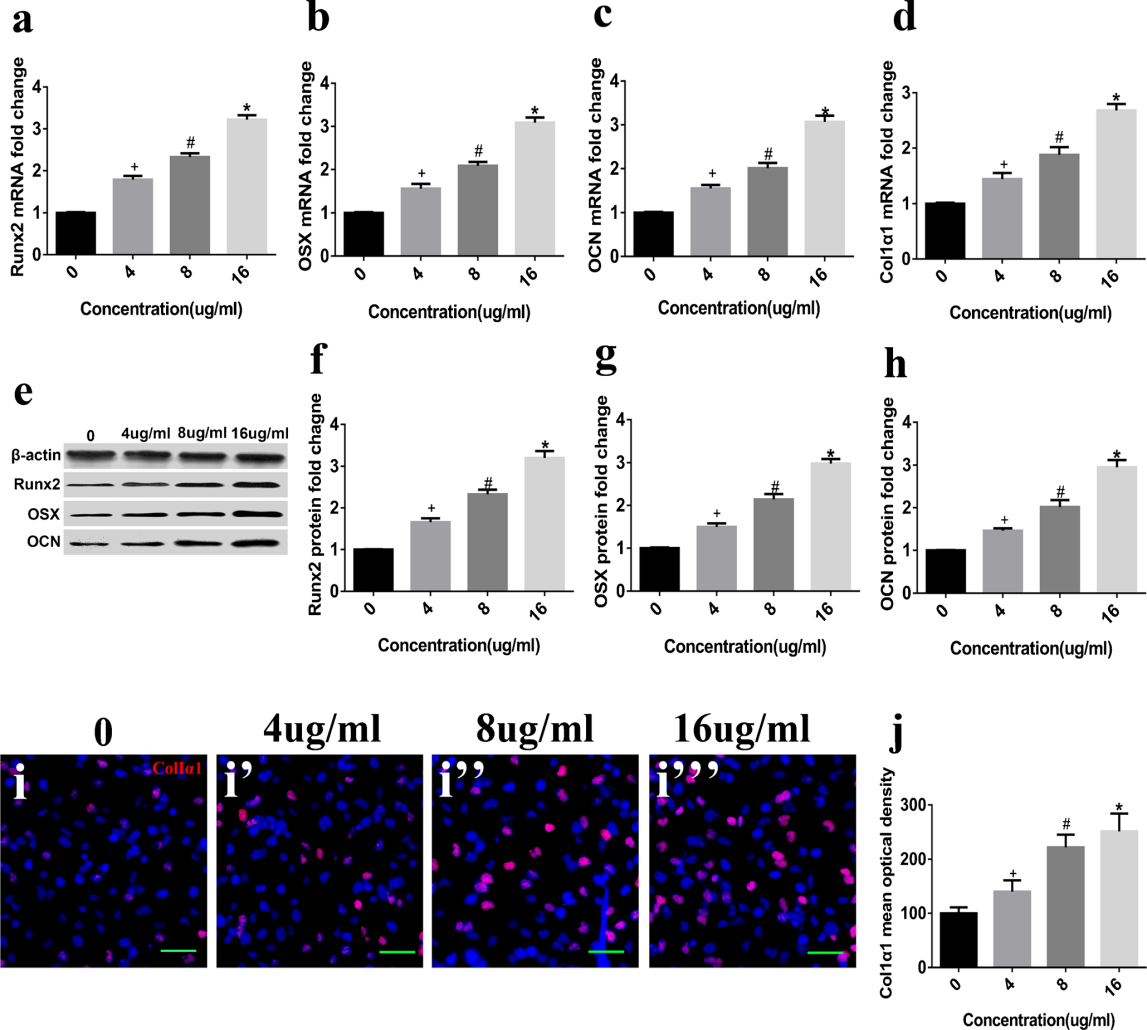
Effect of TEN on osteogenic markers in vitro. BMSCs were cultured in osteogenic medium and exposed to TEN (4, 8, 16 μg/ml) for 14 days. Total cytosolic RNA was prepared and used for quantitative RT polymerase chain reaction analysis of Runx2 (**a**), OSX (**b**), OCN (**c**) and Col Iα1 (**d**) as described in “Materials and methods”. Cell lysates (20 μg) were obtained for western blot and analyzed using antibodies specific for Runx2 (**e**, **f**), OSX (**e**, **g**) and OCN (**e**, **h**). The expression of Col Iα1 was assessed by immunofluorescence staining (**i**–**i”’**, **j**). *Scale bar* 5 μm. *Column*s represent means ± SD from 3 independent experiments, each performed in triplicate

To examine the effects of TEN on osteogenic differentiation, BMSCs were treated with TEN (4, 8 and 16 μg/ml) in osteogenic medium for 14 days. Then, ALP activity/staining and Alizarin red staining were measured and the expression of the osteogenic markers Runx2, OSX, OCN and Col Iα1 was determined by qRT-PCR, WB, or immunostaining. TEN increased the expression of ALP (Fig. 1c, e–e”’), while mineralized nodules (Fig. 1d) and Alizarin red staining (Fig. 1f–f”’) were also improved in a dose-dependent manner. The mRNA expression of Runx2 (Fig. 2a), OSX (Fig. 2b), OCN (Fig. 2c) and Col Iα1 (Fig. 2d) was upregulated in a dose-dependent manner, as shown by qRT-PCR. In addition, the protein expression of Runx2 (Fig. 2e, f), OSX (Fig. 2e, g) and OCN (Fig. 2e, h) was also increased in a dose-dependent manner. Immunostaining for Col Iα1 (Fig. 2i–i”’, j) showed a dose-dependent increase that peaked at 16 μg/ml. These results suggest that TEN promotes in vitro osteogenesis.

### Effect of TEN on femur bone microstructure morphometrics and histologic appearance

**Fig. 3:**
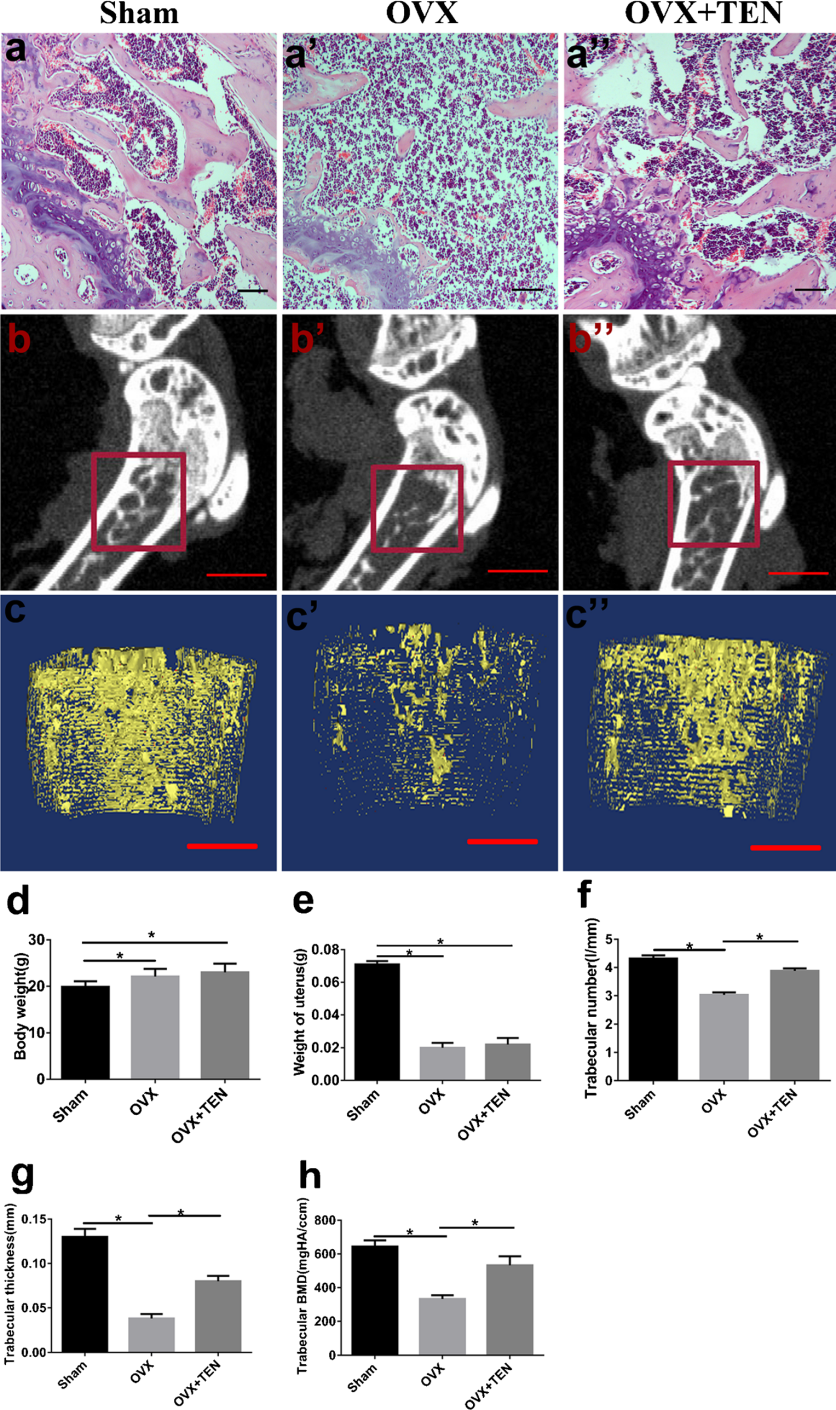
TEN prevents bone loss in OVX mice. **a**–**a”** HE staining of femur sections was assessed after 3 months of treatment. **b**–**b”**, **c**–**c”** The femurs were scanned with a high-resolution X-ray micro-CT and the microstructural indices were performed with the micro-CT data as described in “Materials and methods”, including trabecular number (**f**), trabecular thickness (**g**) and trabecular BMD (**h**). Body weight (**d**) and uterine weight (**e**) were also performed to confirm the effect of the ovarian excision. *Scale bars* (**a**–**a”**) 50 μm, (**b**–**b”**) 1 mm, (**c**–**c”**) 100 μm. Data are shown as mean ± SD from at least three independent experiments, *P < 0.05

To confirm the effect of ovariectomy, body weight and the mass of the uterus were recorded. Body weight increased in the OVX and OVX+TEN groups (Fig. 3d). The mass of the uterus in the sham group was significantly greater than that in the OVX group or OVX+TEN group (Fig. 3e). These data confirm that the excision of the ovaries was successful. To evaluate the effect of TEN on bone formation in mice, we performed X-ray μCT analyses of sham, OVX and OVX+TEN mice. X-ray μCT results for the long bones of the OVX+TEN mice demonstrated a clear trend in which trabecular thickness was increased relative to that in the OVX group (Fig. 3b–b”, c–c”, g). Trabecular number (Fig. 3b–b”, c–c”, f) and trabecular BMD (Fig. 3b–b”, c–c”, h) in bones from the OVX+TEN group were significantly higher than those in bones from the OVX group. However, these parameters were not significantly different between the OVX and sham groups. HE staining of femoral bone sections indicated that the number of trabeculae was significantly lower in OVX mice than in the OVX+TEN group (Fig. 3a-a”).

### TEN promotes the expression of Runx2, OSX and OCN in vivo

**Fig. 4:**
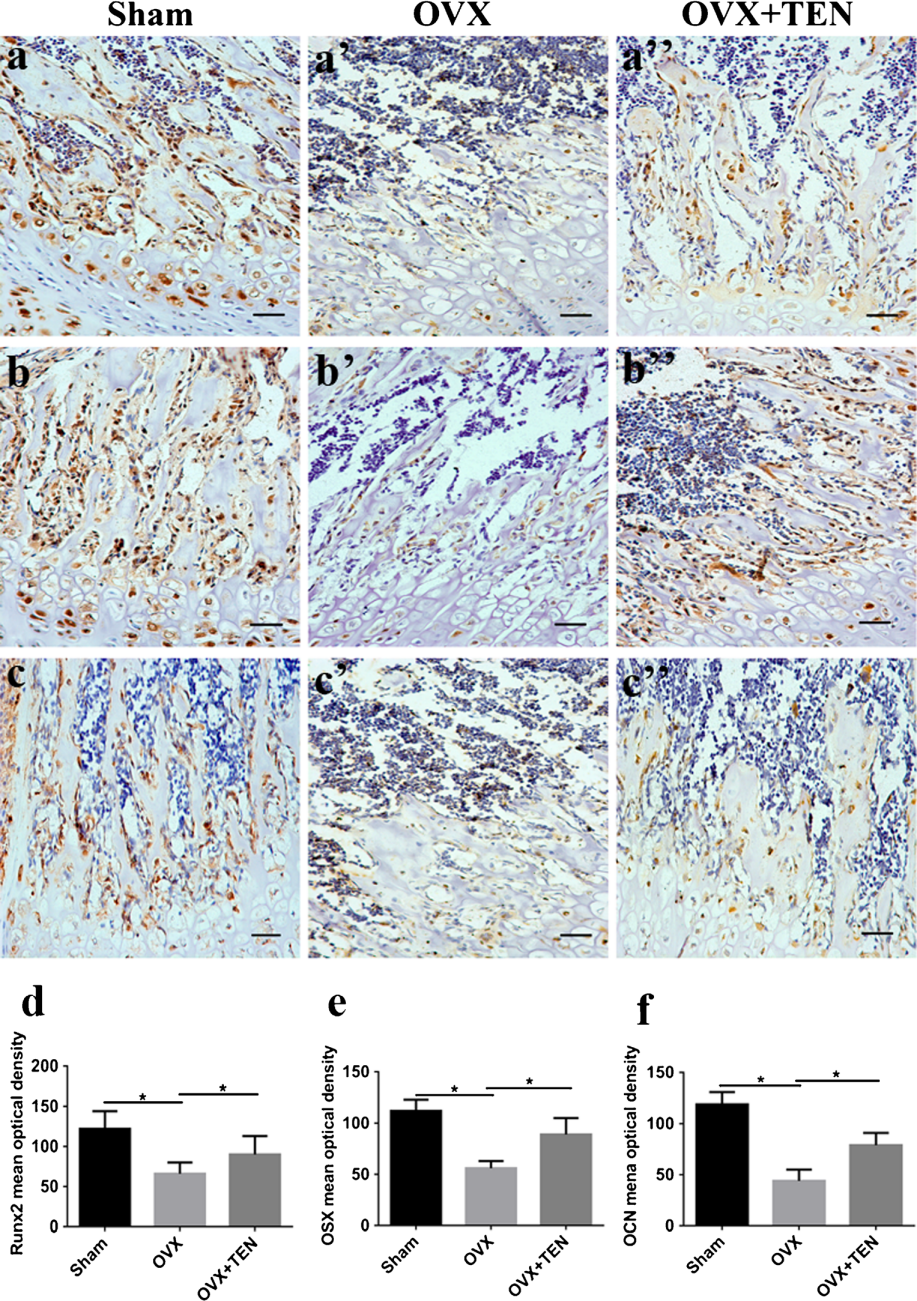
Effect of TEN on the expression of Runx2, OSX and OCN in vivo. Weak positive staining for Runx2 (**a**–**a”**, **d**), OSX (**b**–**b”**, **e**) and OCN (**c**–**c”**, **f**) was observed, especially on the surface of bone lacuna in the OVX group after 3 months. *Scale bars* (**a**–**a”**), (**b**–**b”**), (**c**–**c”**) 20 μm. *Columns* represent means ± SD from at least three independent experiments, *P < 0.05

To examine the molecular and cellular changes associated with TEN-mediated protection against bone loss in vivo, we examined the epiphyseal growth plate, where bone formation occurs on a cartilaginous template (Karsenty 2003; Kronenberg 2003). Expression of Runx2 (Fig. 4a–a”, d), OSX (Fig. 4b–b”, e) and OCN (Fig. 4c–c”, f) was markedly lower in OVX mice than in sham and OVX+TEN mice. However, there were no statistically significant differences between the sham group and the OVX+TEN group. Our in vitro and in vivo data indicate that TEN-induced osteogenesis positively regulates bone formation.

### Effect of TEN on WNT/β-catenin signaling

**Fig. 5:**
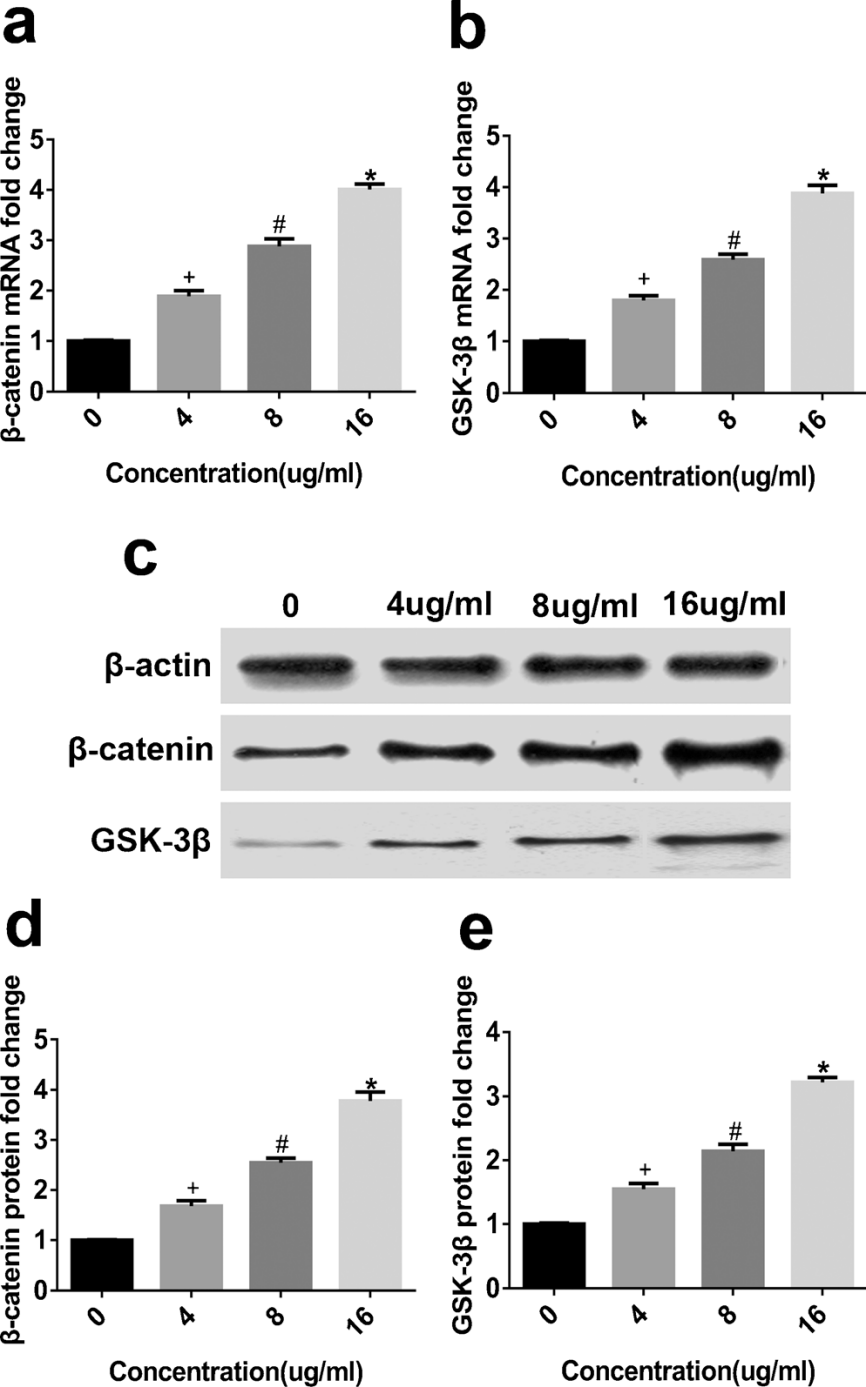
Effect of TEN on WNT/β-catenin signaling. BMSCs were cultured in osteogenic differentiation medium with the treatment of TEN (4, 8, 16 ug/ml) for 14 days. The mRNA expression of β-catenin (**a**) and GSK-3β (**b**) was measured by Q-PCR. The protein expression of β-catenin (**c**, **d**) and GSK-3β (**c**, **e**) was assessed by western blot. ^+^ *P* < 0.05 veruss group without TEN, ^#^ *P* < 0.05 compared with 4 μg/ml TEN, *P < 0.05 compared with 8 μg/ml TEN. *Columns* represent mean ± SD from 3 independent experiments, each performed in triplicate

To gain further insight into the function of TEN in the osteogenic differentiation of cultured BMSCs, we examined the mRNA expression of β-catenin and GSK-3β in vitro. Our qRT-PCR results showed that β-catenin (Fig. 5a) and GSK-3β (Fig. 5b) expression was enhanced. In addition, we investigated the β-catenin (Fig. 5c, d) and GSK-3β (Fig. 5c, e) protein levels, which were both increased in BMSCs after TEN treatment. Collectively, these data indicate that WNT/β-catenin signaling is increased in BMSCs in the presence of TEN and suggest that TEN cooperates with WNT/β-catenin signaling to regulate bone formation.

## Discussion

TEN is the active component of the Chinese herb *Polygala tenuifolia* root that has no genotoxic effects and is safe at the proper dose (Shin et al. 2015). It has been suggested as a potential drug for the treatment of osteoporosis (Yang et al. 2015). TEN also belongs to the family of saponin compounds, including ginsenoside, asperosaponin VI, diosgenin and platycodon, which inhibit osteoclastogenesis and stimulate osteoblast differentiation (Folwarczna et al. 2016; Gu et al. 2016; Jeong et al. 2010; Niu et al. 2011). Based on the reported effect of TEN on the inhibition of osteoclastogenesis, we explored its effect on BMSC osteogenic differentiation and bone formation. The results of the present study showed that TEN promoted osteoblast differentiation, which is similar to data obtained with other saponin compounds. These results suggest that TEN could be a novel compound for the modulation of osteoblast differentiation and indicate a need for continued study of TEN as a potential therapeutic agent for bone disorders, since it is expected to not only enhance bone formation but also suppress bone resorption.

Osteogenic differentiation is a complex process that involves a large number of regulators such as ALP, Ruxn2, OSX, OCN and Col Iα1 (Xu et al. 2012; Yonezawa et al. 2011). ALP, a cell membrane-associated enzyme, appears early during the differentiation of osteoblasts and is the most widely recognized marker of osteoblastic differentiation (Zou et al. 2008; Serigano et al. 2010). ALP activity correlates with matrix formation in osteoblasts prior to the initiation of mineralization (Ying et al. 2013). Runx2 and OSX are the primary regulators of osteogenic differentiation and modulate the expression of other bone markers (Lee et al. 2008). OCN is involved in controlling the mineralization process; it appears at a late stage of osteogenic differentiation and is expressed in mature cells of the osteoblastic lineage (Sun et al. 2010; Hauschka et al. 1989). Col Iα1, an important component of the bone extracellular matrix, connects the cell surface integrins with other extracellular matrix proteins (Lian and Stein 1992). In the present study, TEN effectively upregulated ALP, Runx2, OSX, OCN and Col Iα1, thereby enhancing mineralization. TEN promoted the osteogenic differentiation and maturation of BMSCs, suggesting that TEN is effective in preventing osteoporosis by promoting BMSC function.

To investigate the effects of TEN on postmenopausal osteoporosis in vivo, we used the OVX mice model (Spilmont et al. 2013). Our data showed that TEN significantly improved the trabecular bone microarchitecture of the distal femur in OVX mice. This suggests that TEN is effective in preventing OVX-induced bone loss. These findings were confirmed by histological examination of femur tissues using HE staining and by assessing the expression of Runx2, OSX and OCN in femurs. Consistent with the in vitro results, TEN upregulated osteogenic markers in vivo, indicating that TEN stimulates bone formation and growth. Taken together, our in vitro and in vivo results show that TEN not only promoted osteogenic differentiation in the early stage but also in the late stage of the osteogenic process, which is similar to the effects of other saponin compounds such as ginsenoside, asperosaponin VI, diosgenin and platycodon.

Several studies have reported that osteoporosis in postmenopausal women is often accompanied by depression, neurasthenia and hypomnesis (Gonzalez-Rodriguez et al. 2016; Heuser and Staemmler 1973; Hickey et al. 2016). TEN has been shown to ameliorate learning and memory impairments induced by ovariectomy (Cai et al. 2013). As TEN has anti-aging, anti-depressant, neuroprotective and neuroregenerative effects, it may be more effective than other saponin compounds. TEN is suitable for the treatment of women with osteoporosis associated with depression, neurasthenia, hypomnesis, memory impairments and many other degenerative diseases.

WNT/β-catenin signaling, which is a crucial signal pathway, has sparked great interest in recent decades for its essential role in regulating bone biology and disease (Gu et al. 2015; Manolagas 2014; Tao et al. 2016). A previous study reported that WNT signaling can directly suppress the commitment of MSCs to chondrogenic and adipogenic lineages and enhance osteogenic differentiation via both β-catenin-dependent and β-catenin-independent mechanisms (Hill et al. 2005). Moreover, the expression of the markers of mature osteoblasts ALP, OSX, Col Iα1, Runx2 and OCN in osteoblasts can be increased by activation of the WNT/β-catenin pathway (Frey et al. 2015; Hwang et al. 2015; Li et al. 2015). To gain further insight into TEN-induced osteogenic differentiation, we investigated the expression of β-catenin and GSK3β, which are key factors in WNT signaling that regulate bone formation (Rossini et al. 2013). Our results showed that β-catenin and GSK3β were upregulated by TEN, suggesting that TEN stimulates BMSC osteogenic differentiation by activating WNT signaling.

The present study had several limitations. First, the effect of TEN on rat BMSCs was examined under osteogenic differentiation conditions. The effect of TEN on human BMSCs needs to be examined to substantiate these results. Second, although our findings indicate a protective effect of TEN on osteoporotic bone, we did not include a positive control group, such as estrogen treatment or a control+TEN group. A control group, control+TEN group and sham+estrogen group are worthy of further in-depth study. Third, we only obtained BMSCs from mice because of ethical reasons. Additional human BMSCs from healthy persons and osteoporosis patients would be helpful to identify whether these findings are specific.

**Fig. 6:**
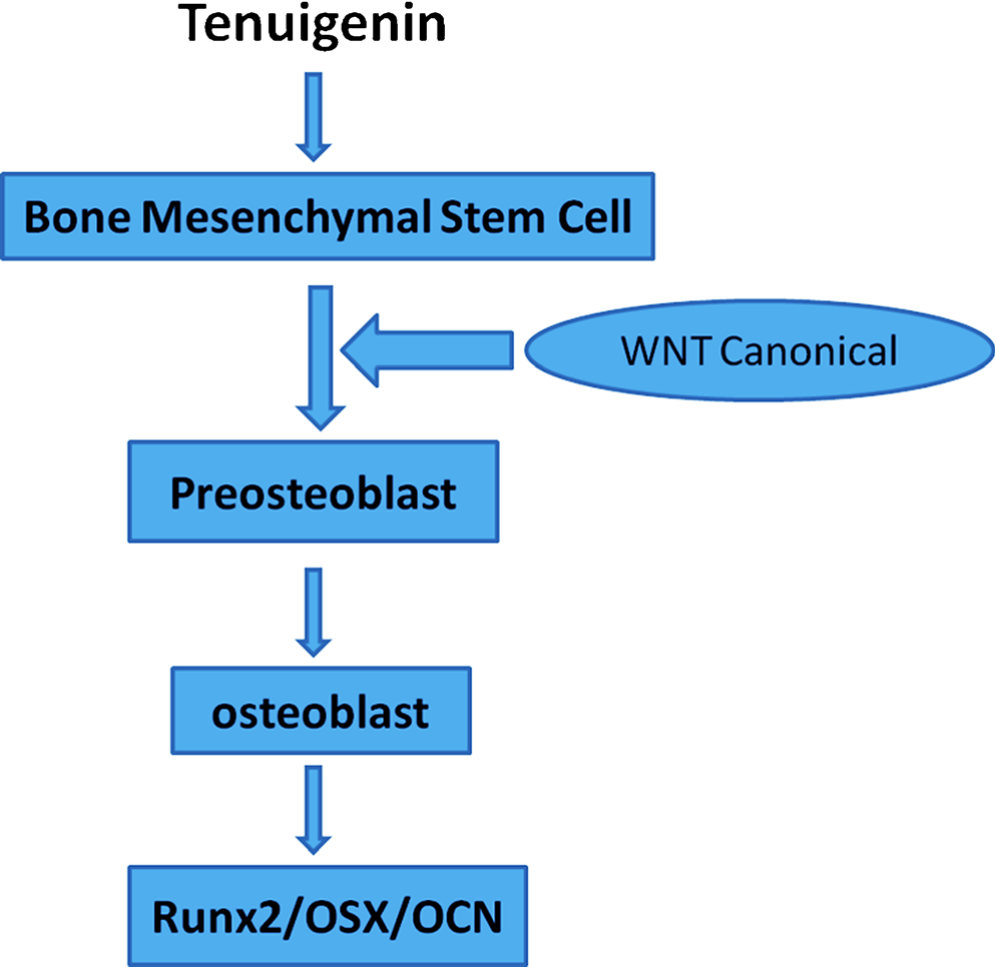
TEN promotes differentiation of BMSCs into osteoblasts. The maturation and survival of osteoblasts are supported by WNT canonical signaling, resulting in the enhancement of Runx2, OSX and OCN

In conclusion, our study provides novel insights into the effects of TEN on BMSC osteogenic differentiation and its protective effect on bone loss in mice. We demonstrated, for the first time, that TEN promotes osteogenesis by upregulating Runx2, OSX and OCN at the tissue and cellular levels, thereby promoting the lineage differentiation of BMSCs into osteoblasts. Furthermore, we have shown that WNT signaling is at least partly involved as an underlying mechanism in this process (Fig. 6). In future studies, the use of TEN for the stimulation of bone formation in postmenopausal women and the treatment of depression, neurasthenia, hypomnesis, memory impairments and many other degenerative diseases should be examined by performing pharmacokinetic and toxicological analyses in humans as well as randomized control studies.

## References

[CR1] Bagan J, Peydro A, Calvo J, Leopoldo M, Jimenez Y, Bagan L (2016). Medication-related osteonecrosis of the jaw associated with bisphosphonates and denosumab in osteoporosis. Oral Dis.

[CR2] Bianco P, Kuznetsov SA, Riminucci M, Gehron RP (2006). Postnatal skeletal stem cells. Methods Enzymol.

[CR3] Boyle WJ, Simonet WS, Lacey DL (2003). Osteoclast differentiation and activation. Nature.

[CR4] Cai ZL, Wang CY, Gu XY, Wang NJ, Wang JJ, Liu WX, Xiao P, Li CH (2013). Tenuigenin ameliorates learning and memory impairments induced by ovariectomy. Physiol Behav.

[CR5] Demontiero O, Vidal C, Duque G (2012). Aging and bone loss: new insights for the clinician. Ther Adv Musculoskelet Dis.

[CR6] Eriksen EF, Diez-Perez A, Boonen S (2014). Update on long-term treatment with bisphosphonates for postmenopausal osteoporosis: a systematic review. Bone.

[CR7] Folwarczna J, Zych M, Nowinska B, Pytlik M, Bialik M, Jagusiak A, Lipecka-Karcz M, Matysiak M (2016). Effect of diosgenin, a steroidal sapogenin, on the rat skeletal system. Acta Biochim Pol.

[CR8] Frey JL, Li Z, Ellis JM, Zhang Q, Farber CR, Aja S, Wolfgang MJ, Clemens TL, Riddle RC (2015). Wnt-Lrp5 signaling regulates fatty acid metabolism in the osteoblast. Mol Cell Biol.

[CR9] Gonzalez-Rodriguez, A., Catalan, R., Penades, R., and Bernardo, M. (2016). The oestrogen dysfunction hypothesis in schizophrenia: The need for an integrative approach to treat postmenopausal women. Aust N Z J Psychiatry (in press) 10.1177/0004867416649033 27207861

[CR10] Gu Q, Chen C, Zhang Z, Wu Z, Fan X, Zhang Z, Di W, Shi L (2015). Ginkgo biloba extract promotes osteogenic differentiation of human bone marrow mesenchymal stem cells in a pathway involving Wnt/beta-catenin signaling. Pharmacol Res.

[CR11] Gu Y, Zhou J, Wang Q, Fan W, Yin G (2016). Ginsenoside Rg1 promotes osteogenic differentiation of rBMSCs and healing of rat tibial fractures through regulation of GR-dependent BMP-2/SMAD signaling. Sci Rep.

[CR12] Harada S, Rodan GA (2003). Control of osteoblast function and regulation of bone mass. Nature.

[CR13] Hauschka PV, Lian JB, Cole DE, Gundberg CM (1989). Osteocalcin and matrix Gla protein: vitamin K-dependent proteins in bone. Physiol Rev.

[CR14] Heuser HP, Staemmler HJ (1973). Histological investigations into the effect of oestriol succinate on the corpus uteri in postmenopausal women. Arzneimittelforschung.

[CR15] Hickey, M., Schoenaker, D.A., Joffe, H., and Mishra, G.D. (2016). Depressive symptoms across the menopause transition: findings from a large population-based cohort study. Menopause (in press) 10.1097/GME.0000000000000712 27552471

[CR16] Hill TP, Spater D, Taketo MM, Birchmeier W, Hartmann C (2005). Canonical Wnt/beta-catenin signaling prevents osteoblasts from differentiating into chondrocytes. Dev Cell.

[CR17] Hwang JH, Cha PH, Han G, Bach TT, Min DS, Choi KY (2015). Euodia sutchuenensis Dode extract stimulates osteoblast differentiation via Wnt/beta-catenin pathway activation. Exp Mol Med.

[CR18] Jeong HM, Han EH, Jin YH, Hwang YP, Kim HG, Park BH, Kim JY, Chung YC, Lee KY, Jeong HG (2010). Saponins from the roots of Platycodon grandiflorum stimulate osteoblast differentiation via p38 MAPK- and ERK-dependent RUNX2 activation. Food Chem Toxicol.

[CR19] Karsenty G (2003). The complexities of skeletal biology. Nature.

[CR20] Kronenberg HM (2003). Developmental regulation of the growth plate. Nature.

[CR21] Lee HW, Suh JH, Kim HN, Kim AY, Park SY, Shin CS, Choi JY, Kim JB (2008). Berberine promotes osteoblast differentiation by Runx2 activation with p38 MAPK. J Bone Miner Res.

[CR22] Li X, Lim J, Lu J, Pedego TM, Demer L, Tintut Y (2015). Protective Role of Smad6 in Inflammation-Induced Valvular Cell Calcification. J Cell Biochem.

[CR23] Li F, Zhou C, Xu L, Tao S, Zhao J, Gu Q (2016) Effect of Stem Cell Therapy on Bone Mineral Density: A Meta-Analysis of Preclinical Studies in Animal Models of Osteoporosis. PLoS ONE 11, e149400 10.1371/journal.pone.0149400 PMC4755606 26882451

[CR24] Lian JB, Stein GS (1992). Concepts of osteoblast growth and differentiation: basis for modulation of bone cell development and tissue formation. Crit Rev Oral Biol Med.

[CR25] Liang Z, Shi F, Wang Y, Lu L, Zhang Z, Wang X, Wang X (2011). Neuroprotective effects of tenuigenin in a SH-SY5Y cell model with 6-OHDA-induced injury. Neurosci Lett.

[CR26] Liu W, Yang LH, Kong XC, An LK, Wang R (2015). Meta-analysis of osteoporosis: fracture risks, medication and treatment. Minerva Med.

[CR27] Luhe A, Kunkele KP, Haiker M, Schad K, Zihlmann C, Bauss F, Suter L, Pfister T (2008). Preclinical evidence for nitrogen-containing bisphosphonate inhibition of farnesyl diphosphate (FPP) synthase in the kidney: implications for renal safety. Toxicol In Vitro.

[CR28] Ma ZP, Liao JC, Zhao C, Cai DZ (2015). Effects of the 1, 4-dihydropyridine L-type calcium channel blocker benidipine on bone marrow stromal cells. Cell Tissue Res.

[CR29] Manolagas SC (2014). Wnt signaling and osteoporosis. Maturitas.

[CR30] Niu Y, Li Y, Huang H, Kong X, Zhang R, Liu L, Sun Y, Wang T, Mei Q (2011). Asperosaponin VI, a saponin component from Dipsacus asper wall, induces osteoblast differentiation through bone morphogenetic protein-2/p38 and extracellular signal-regulated kinase 1/2 pathway. Phytother Res.

[CR31] Rodan GA, Martin TJ (2000). Therapeutic approaches to bone diseases. Science.

[CR32] Rossini M, Gatti D, Adami S (2013). Involvement of WNT/beta-catenin signaling in the treatment of osteoporosis. Calcif Tissue Int.

[CR33] Safer, U., Safer, V.B., Demir, S.O., and Yanikoglu, I. (2016). Effects of Bisphosphonates and Calcium plus Vitamin-D Supplements on Cognitive Function in Postmenopausal Osteoporosis. Endocr Metab Immune Disord Drug Targets (in press) 10.2174/1871530316666160330105952 27026340

[CR34] Serigano K, Sakai D, Hiyama A, Tamura F, Tanaka M, Mochida J (2010). Effect of cell number on mesenchymal stem cell transplantation in a canine disc degeneration model. J Orthop Res.

[CR35] Shin KY, Won BY, Ha HJ, Yun YS, Lee HG (2015). Genotoxicity studies on the root extract of Polygala tenuifolia Willdenow. Regul Toxicol Pharmacol.

[CR36] Spilmont M, Leotoing L, Davicco MJ, Lebecque P, Mercier S, Miot-Noirault E, Pilet P, Rios L, Wittrant Y, Coxam V (2013). Pomegranate seed oil prevents bone loss in a mice model of osteoporosis, through osteoblastic stimulation, osteoclastic inhibition and decreased inflammatory status. J Nutr Biochem.

[CR37] Sun GB, Deng XC, Li CH (2007). The protective effects of tenuigenin on the PC12 cells injury induced by H2O2. Zhong Yao Cai.

[CR38] Sun H, Feng K, Hu J, Soker S, Atala A, Ma PX (2010). Osteogenic differentiation of human amniotic fluid-derived stem cells induced by bone morphogenetic protein-7 and enhanced by nanofibrous scaffolds. Biomaterials.

[CR39] Tao K, Xiao D, Weng J, Xiong A, Kang B, Zeng H (2016). Berberine promotes bone marrow-derived mesenchymal stem cells osteogenic differentiation via canonical Wnt/beta-catenin signaling pathway. Toxicol Lett.

[CR40] van den Bergh JP, van Geel TA, Geusens PP (2012). Osteoporosis, frailty and fracture: implications for case finding and therapy. Nat Rev Rheumatol.

[CR41] Xu YX, Wu CL, Wu Y, Tong PJ, Jin HT, Yu NZ, Xiao LW (2012). Epimedium-derived flavonoids modulate the balance between osteogenic differentiation and adipogenic differentiation in bone marrow stromal cells of ovariectomized rats via Wnt/beta-catenin signal pathway activation. Chin J Integr Med.

[CR42] Yang S, Li X, Cheng L, Wu H, Zhang C, Li K (2015). Tenuigenin inhibits RANKL-induced osteoclastogenesis by down-regulating NF-kappaB activation and suppresses bone loss in vivo. Biochem Biophys Res Commun.

[CR43] Ying X, Sun L, Chen X, Xu H, Guo X, Chen H, Hong J, Cheng S, Peng L (2013). Silibinin promotes osteoblast differentiation of human bone marrow stromal cells via bone morphogenetic protein signaling. Eur J Pharmacol.

[CR44] Yonezawa T, Lee JW, Hibino A, Asai M, Hojo H, Cha BY, Teruya T, Nagai K, Chung UI, Yagasaki K (2011). Harmine promotes osteoblast differentiation through bone morphogenetic protein signaling. Biochem Biophys Res Commun.

[CR45] Yu GY, Zheng GZ, Chang B, Hu QX, Lin FX, Liu DZ, Wu CC, Du SX, Li XD (2016). Naringin Stimulates Osteogenic Differentiation of Rat Bone Marrow Stromal Cells via Activation of the Notch Signaling Pathway. Stem Cells Int.

[CR46] Zhang LY, Xue HG, Chen JY, Chai W, Ni M (2016). Genistein induces adipogenic differentiation in human bone marrow mesenchymal stem cells and suppresses their osteogenic potential by upregulating PPARgamma. Exp Ther Med.

[CR47] Zou L, Zou X, Chen L, Li H, Mygind T, Kassem M, Bunger C (2008). Multilineage differentiation of porcine bone marrow stromal cells associated with specific gene expression pattern. J Orthop Res.

